# Characterization of Human Papillomavirus Type 154 and Tissue Tropism of Gammapapillomaviruses

**DOI:** 10.1371/journal.pone.0089342

**Published:** 2014-02-13

**Authors:** Agustín Enrique Ure, Ola Forslund

**Affiliations:** Department of Laboratory Medicine, Section of Medical Microbiology, Lund University, Malmö, Sweden; Albert Einstein College of Medicine, United States of America

## Abstract

The novel human papillomavirus type 154 (HPV154) was characterized from a wart on the *crena ani* of a three-year-old boy. It was previously designated as the putative HPV type FADI3 by sequencing of a subgenomic FAP amplicon. We obtained the complete genome by combined methods including rolling circle amplification (RCA), genome walking through an adapted method for detection of integrated papillomavirus sequences by ligation-mediated PCR (DIPS-PCR), long-range PCR, and finally by cloning of four overlapping amplicons. Phylogenetically, the HPV154 genome clustered together with members of the proposed species *Gammapapillomavirus 11*, and demonstrated the highest identity in L1 to HPV136 (68.6%). The HPV154 was detected in 3% (2/62) of forehead skin swabs from healthy children. In addition, the different detection sites of 62 gammapapillomaviruses were summarized in order to analyze their tissue tropism. Several of these HPV types have been detected from multiple sources such as skin, oral, nasal, and genital sites, suggesting that the gammapapillomaviruses are generalists with a broader tissue tropism than previously appreciated. The study expands current knowledge concerning genetic diversity and tropism among HPV types in the rapidly growing gammapapillomavirus genus.

## Introduction

The papillomaviruses (PVs) are small viruses with icosahedral symmetry and a circular, double stranded genome [1]. These viruses are widely distributed across vertebrates, and among humans alone, more than 170 unique complete genomes of types have so far been sequenced [2]. The papillomaviruses are epitheliotropic and produce hyperproliferation of squamous cells known as papillomas. Human papillomaviruses (HPV) are divided into high-risk types, which are etiologically associated with cancer of the cervix uteri; and low-risk types, which produce benign warts [3], [4]. Regarding the taxonomy of papillomaviruses, the International Committee for the Taxonomy of Viruses (ICTV) recommends inclusion of both genotypic and phenotypic information in the definition of viral genera and species [5]. Nevertheless, papillomaviruses have been an exception to the classical rules, and a system based mainly on sequence identity was adopted (reviewed in de Villiers, 2013). Accordingly, a 70% sequence identity amongst L1 ORFs is used as cut-off guide to classify viruses as belonging to the same species [6]. Even so, there is a gray zone (67.5%–70.5% identity) where the distribution curves for inter- and intraspecies identities overlap, and consequently the classification must be curated [7]. The papillomaviruses have in the past been classified as mucosal or cutaneous according to their tropism. The human cutaneotropic papillomaviruses are represented mainly by the genera *Beta-* and *Gammapapillomavirus* (β-PV and γ-PV). Their prevalence and natural history, for example acquisition soon after birth, reflects a commensalic interaction with the immunocompetent host [8]–[10]. Historically, several cutaneous papillomaviruses have been isolated from patients with the genetic disease epidermodysplasia verruciformis [11], [12], and from immunosuppressed patients [13], probably due to higher viral loads among these patient groups [14]. As a consequence of improved methods, several PVs have been characterized, and the number of HPV types of the γ-PV genus has been expanded from 16 HPV types in 2010 [7] to 62 representative HPV types in 2013 (retrieved from GenBank, September 2013). A review of the isolation sources of these HPV types could lead to increased knowledge of their tropism. Here, we categorized the different isolation sites of the γ-PV as genital, oral, nasal or cutaneous (including skin lesions and healthy skin). The aim of the study was to obtain the complete sequence of a novel papillomavirus, and to present a summary of the isolation sources of the rapidly growing genus *Gammapapillomavirus*.

## Results

HPV154 was isolated from a wart on the *crena ani* of a three-year-old boy. The index sample was negative for HPV by PCR using MGP primers [15] and a Luminex system [16], but positive for HPV by FAP-PCR [10]. The FAP amplicon was sequenced and showed the highest identity to the FADI3 fragment (99.3%, acc. no. FJ480954). The FADI3 was originally amplified from a forehead swab of a six-year-old girl [17].

**Figure 1:**
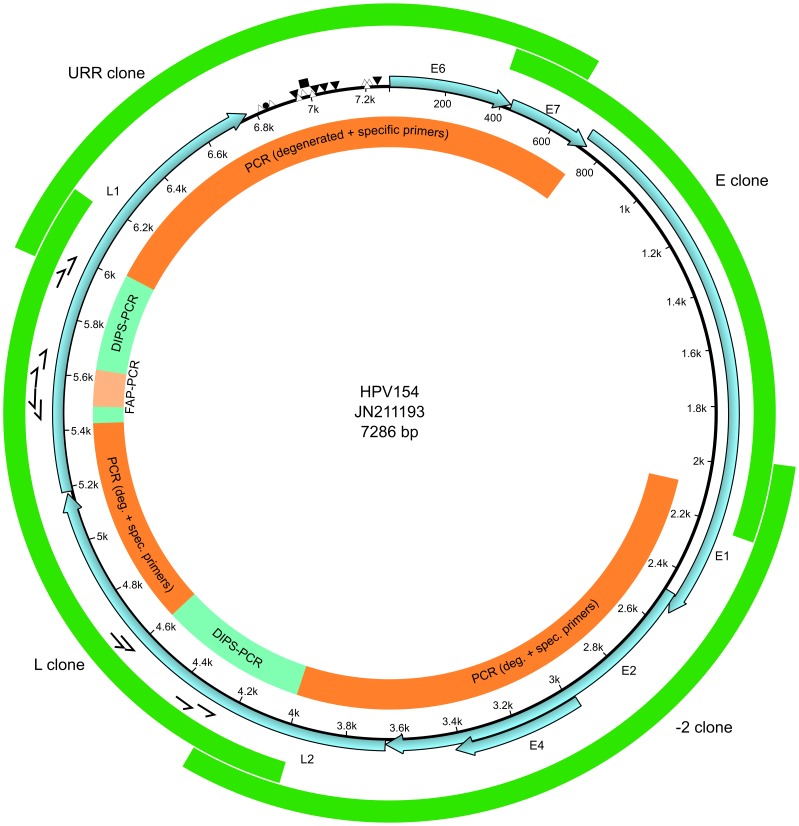
Methodological strategy and genomic organization for HPV154. The ORFs are indicated with light blue arrows. The primers used in the DIPS-PCR are represented with half arrows. The inner circle shows the strategies employed, and the outer circle shows the final clones. Putative binding sites for viral proteins and cellular factors are shown as follows: E2 binding site, E2BS (▾); E1 binding site, E1BS (Δ); TATA-box (▪); Polyadenylation signal (•).

The FAP amplicon represented a putative novel type, as the partial L1 ORF was below 90% of sequence identity to any other known PV type [6]. With or without pre-amplification by rolling circle amplification (RCA), we failed to amplify the complete genome by long-range PCR or with PCRs designed to cover half the genome by a combination of L1-specific primers with degenerated E1 primers (data not shown). As a consequence, we adapted the method for detection of integrated papillomavirus sequences by ligation-mediated PCR (DIPS-PCR) [18] to obtain sequence information outside the FAP amplicon region of the L1 ORF. In order to test the performance of the DIPS-PCR method, we verified the sequence around the integration site of HPV16 into the genome of SiHa cells (data not shown). Several DIPS-PCRs and PCR with degenerated HPV primers were used to obtain sequence data from L2 to almost the end of L1 (Figure 1). Proximal to the ends of that region, new primers were designed that were combined with degenerated primers based on related γ-PVs, and two amplicons of ∼2000 bp were cloned and sequenced. In order to obtain the complete genome, four overlapping amplicons were obtained by PCR with specific primers (Figure 1). The viral load of HPV154 was 68 genomes per human cell (Table 1).

**Table 1:** Characteristics of HPV154 positive children and viral loads in swab samples.

Sample ID	Age	Gender	Swab sampling site	Sample type	Viral genomes/µL (CV%)‡	Viral genomes/human cell (CV%)‡
1*	3	Male	Wart, crena ani	Extracted DNA†	48 (6.3)	68 (7.7)¥
2	3	Male	Wart, crena ani	Saline	41 (20)	39 (95)
3§	1	Female	Forehead	Saline	0.49 (73)	0.093 (59)
4§	4	Female	Forehead	Saline	0.68 (69)¶	2.0 (23)¶

*Index sample used for characterization of HPV154.

† DNA was extracted from sample no. 2.

‡ Viral load measurements were performed in triplicate.

¥ Number of human cells obtained in duplicate due to one unsuccessful measurement.

§ HPV154 detected in 2 forehead swab samples from 62 children.

¶ Number of HPV154 copies obtained in duplicate due to one unsuccessful measurement.

The four clones were submitted to the International Reference Center for Human Papillomaviruses at the German Cancer Research Center, Heidelberg, Germany, and the compiled sequence was verified and officially designated as HPV154 (GenBank JN211193).

The genome of HPV154 comprised 7286 bp with a GC content of 37.9%. The most closely related type was HPV136, isolated from the oral cavity [19], with 68.6% identity at the L1 ORF, whereas the uncloned HPV isolate KN3, obtained by high throughput sequencing from healthy skin [20], showed 71.8% identity at L1.

HPV154 demonstrated the classical genomic organization of PVs, with seven ORFs identified (Figure 1). The putative E6 protein had two zinc-finger domains (CX_2_CX_29/30_CX_2_C) that were separated by 36 amino acids, which are conserved among PVs [21]. Similarly, there was one zinc-finger domain in the inferred E7 protein, as well as the tumor suppressor (pRB) binding domain (LXCXE) [22], [23]. In the putative E1 protein, the superfamily 3 ATP-dependent helicase domain was identified [24], [25]. The theoretical E2 protein had the typical C-terminal DNA-binding domain and the N-terminal trans-activation domain [26], [27]. The E4 ORF showed a start codon; nevertheless it was ignored as we identified the characteristic donor (AAG/GUASNR) and acceptor (GUYACYAG/YU) RNA splicing sites [28], and the resulting putative E1∧E4 fusion protein with six amino acids from the E1 N-terminal end.

The early polyadenylation site (AATAAA) for processing of early mRNAs, was located at the 5′ end of the L2 ORF, while the late polyadenylation site was downstream of L1 within the upstream regulatory region (URR). The URR was relatively short, being 517 bp, and contained six putative E2 binding sites (E2BS, ACCN_6_GGT). Near the E2BS proximal to E6, several E1 binding sites (AACAAT) or related AT-rich sequences were identified and probably represent the origin of replication [29], [30]. A TATA box was found (pos. 6973–6977) and surrounded by two E2BS in close proximity (2 and 14 bp).

As the FADI3 amplicon and HPV154 were obtained from samples from children, we tested whether this PV type might have an increased presence in this group. Among the cutaneous swab samples, HPV154 was detected in 3% (2/62) by real-time PCR. One of the samples showed a viral load of 0.093 genomes and the other 1.3 genomes per human cell (Table 1).

**Figure 2:**
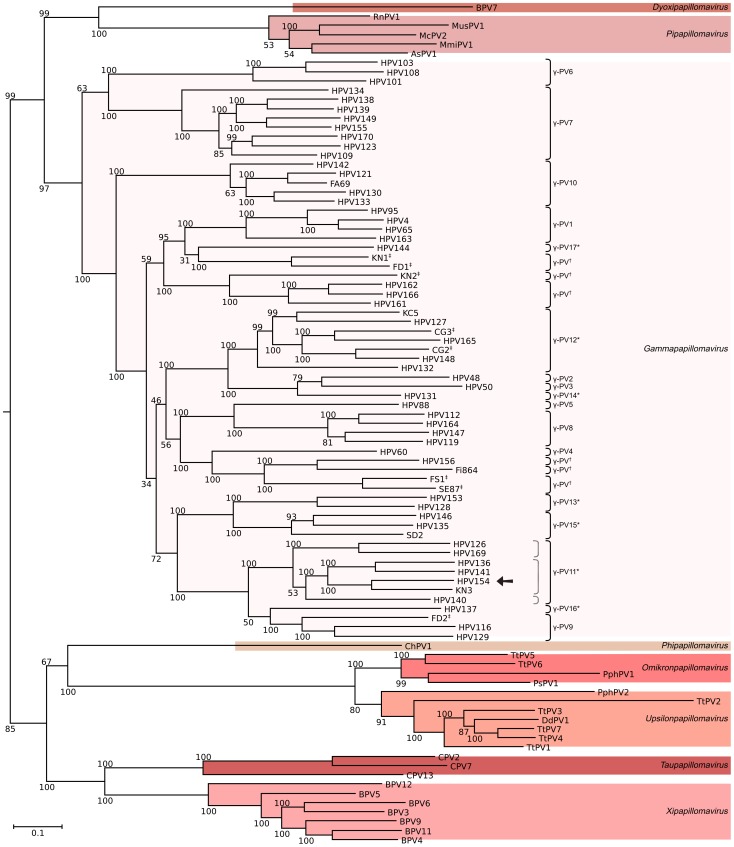
Phylogenetic tree for HPV154 and related papillomaviruses. Ninety one complete genomes were analyzed, including all gammapapillomaviruses as well as related genera but closer than betapapillomaviruses. HPV154 is indicated with an arrow. The tree was obtained by the maximum likelihood approach using RAxML software and rooted with the betapapillomavirus HPV5 and HPV9. Bootstrap support values are indicated in each branch as percentages. Genera are indicated at the rightmost side, while species of γ-PVs are to the right of brackets. The dotted key to the left of γ-PV11* shows species suggested in this work. The bar indicates the number of substitutions per site. * Proposed category and pending approval by ICTV. †Putative novel species. ‡: Uncloned HPV genome (not official type). The following species are represented by the types included in the tree: AsPV1, *Apodemus sylvaticus papillomavirus 1*; BPV, *Bos taurus papillomavirus spp.*; ChPV1, *Capra hircus papillomavirus 1*; CPV, *Canis familiaris papillomavirus spp.*; DdPV1, *Delphinus delphis papillomavirus 1*; HPV, *Gammapapillomavirus spp.*; McPV2, *Mastomys coucha Papillomavirus 2*; PphPV, *Phocoena phocoena papillomavirus spp.*; PsPV1, *Phocoena spinipinnis Papillomavirus 1*; TtPV, *Tursiops truncatus Papillomavirus spp*.

The phylogenetic relationships of HPV154 were inferred based on the complete genomes of 91 HPV-sequences (Figure 2). HPV154 was positioned on a divergent group, along with the members of the currently proposed γ-PV11 species (pending ICTV approval) (Figure 2). HPV154 is below the 70% identity limit suggested for species definition compared to its closest relative type, HPV136 (68.6%). Moreover, the lowest values of identity for HPV154 within γ-PV11 (Figure 2) are with HPV140 (65.37%) and HPV169 (65.86%).

In order to increase knowledge of the biological niches of gammapapillomaviruses, we enumerated the isolation sources (complete HPV-genomes) and detection sites of all HPV types from this genus (Table 2). The γ-PV1 species contains members isolated from a wide variety of sites, including healthy tissue such as the skin, nasal cavity and male genitalia. However, they are also found in non-healthy tissue such as common warts, pigmented verrucas, squamous cell carcinoma (SCC) and actinic keratosis (AK). In a similar fashion, HPV types of the γ-PV3, γ-PV7, γ-PV8, γ-PV9, γ-PV10, γ-PV11 and γ-PV15 species have been found in several different sites, including both mucosal and cutaneous sources (Table 2). The γ-PV6 members (HPV101, 103 and 108) are the only γ-PV restricted to genital and oral mucosal sites (six isolates). On the other hand, the γ-PV12 members have been isolated only from warts and healthy skin (eight isolates). The remaining species, which include γ-PV2, γ-PV4, γ-PV5, γ-PV13, γ-PV14, γ-PV16, γ-PV17 and the six putative novel species (Figure 2), have members with less than three findings, but include both mucosal and cutaneous sites (Table 2).

**Table 2:** Sites of detection of gammapapillomaviruses.

HPV type	Species	Site	References	FA-fragment
				(% identity to type)
HPV4	γ-PV1	Common wart	[64]	
"	''	Male genitalia	[57]	
''	''	Nasal cavity	[39]	
''	''	Skin	[62]	
HPV65	γ-PV1	Pigmented verruca	[69]	
''	''	SCC/AK	[38]	
HPV95	γ-PV1	Skin wart	[71]	
HPV48	γ-PV2	SCC	[72]	
HPV50	γ-PV3	Skin macules EV	[56]	
''	''	Nasal cavity	[39]	
''	''	Skin	[9], [61], [62]	
''	''	SCC/AK and keratoacanthoma	[38]	
HPV60	γ-PV4	Planta cyst	[63]	
HPV88	γ-PV5	Skin wart	[36]	
HPV101	γ-PV6	Cervicovaginal	[42]	
''	''	Male genitalia	[57]	
HPV103	γ-PV6	Cervicovaginal	[42]	
''	''	Male genitalia	[57]	
''	''	Nasal cavity	[39]	
HPV108	γ-PV6	Cervicovaginal	[43]	
HPV109	γ-PV7	SCC	[37]	
''	''	Seborrhoeic keratosis	[37]	
''	''	Cervicovaginal (CINI)	[37]	
''	''	SCC	[65]	FA137 (92.7)
HPV123	γ-PV7	Oral cavity	[19]	
''	''	Skin SCC/AK	[38]	
''	''	Skin Seborheic keratosis	[65]	FA136 (94.7)
HPV134	γ-PV7	Oral cavity	[19]	
''	''	Male genitalia	[57]	
''	''	Nasal cavity	[39]	
''	''	AK	[10]	FA1.1 (94.6)
''	''	Skin	[10]	FA1.1 (94.6)
HPV138	γ-PV7	Oral cavity	[19]	
''	''	Nasal cavity	[39]	
''	''	Skin	[8]	FA20 (91.5)
HPV139	γ-PV7	Oral cavity	u. d.	
''	''	Cutaneous adnexal tumour	[66]	FA105 (92.2)
HPV149	γ-PV7	Skin wart	[13]	
HPV155	γ-PV7	Actinic keratosis	[38]	
''	''	Nasal cavity	[39]	FA166 (91.4)
HPV170	γ-PV7	Skin	[59]	
HPV112	γ-PV8	Condyloma acuminata	[37]	
HPV119	γ-PV8	Oral cavity	[19]	
''	''	Male genitalia	[57]	
''	''	Keratoacanthoma	[38]	
''	''	Skin	[67]	FA155 (90.8)
HPV147	γ-PV8	Oral cavity	u. d.	
''	''	Nasal cavity	[39]	
''	''	Skin	[10]	FA13 (94.7)
HPV164	γ-PV8	Skin	[59]	
HPV116	γ-PV9	Rectal Swab	[68]	
HPV129	γ-PV9	Skin wart	[13]	
FD2‡	''	Skin	u. d.	
HPV121	γ-PV10	Oral cavity	[19]	
''	''	Male genitalia	[57]	
HPV130	γ-PV10	Skin wart	[13]	
''	''	Male genitalia	[57]	
''	''	Nasal cavity	[39]	
''	''	Actinic keratosis	u. d.	FA147 (95.1)
HPV133	γ-PV10	Skin wart	[13]	
''	''	Male genitalia	[57]	
''	''	Nasal cavity	[39]	
''	''	Skin	[61]	FA44 (92.4)
HPV142	γ-PV10	Oral cavity	u. d.	
''	''	Nasal cavity	[39]	
''	''	Skin	[9]	FA67 (92.2)
FA69‡	γ-PV10	Condyloma acuminata	[60]	
''	''	Skin	[9]	FA69 (100)
HPV126	γ-PV11*	Skin wart	[70]	
HPV136	γ-PV11*	Oral cavity	[19]	
''	''	Skin	[10]	FA8 (91.8)
HPV140	γ-PV11*	Oral cavity	u. d.	
HPV141	γ-PV11*	Oral cavity	u. d.	
HPV154	γ-PV11*	Skin wart	Present study	
''	''	Skin	[17]	FADI3 (91.3)
KN3‡		Skin	u. d.	
HPV169	γ-PV11*	Skin	[59]	
HPV132	γ-PV12*	Skin wart	[13]	
''	''	Skin	[61]	FA78 (91.3)
HPV148	γ-PV12*	Skin wart	[13]	
HPV165	γ-PV12*	Skin	[59]	
CG2‡	γ-PV12*	Skin	[20]	
''	''	Skin	[66]	FA106 (96.2)
CG3†	γ-PV12*	Skin	[20]	
KC5‡	''	Skin	[59]	
HPV128	γ-PV13*	Skin wart	[13]	
HPV153	γ-PV13*	Condyloma acuminata	[44]	
HPV131	γ-PV14*	Skin wart	[13]	
HPV135	γ-PV15*	Oral cavity	[19]	
''	''	Male genitalia	[57]	
''	''	Nasal cavity	[39]	
''	''	Skin	[8]	FA28 (97.5)
HPV146	γ-PV15*	Oral cavity	u. d.	
''	''	Nasal cavity	[39]	
''	''	Skin	[9]	FA35 (96.8)
'' CG1‡		Skin	[20]	
SD2‡		Nasopharyngeal and oropharyngeal	[73]	
HPV137	γ-PV16*	Oral cavity	[19]	
HPV144	γ-PV17*	Oral cavity	[19]	
''	''	Male genitalia	[57]	
HPV156	γ-PV†	Skin	[58]	
Fi864‡	γ-PV†	Nasal swab	[40]	
HPV161	γ-PV†	Skin	[59]	
HPV162	''	Skin	[59]	
HPV166	''	Skin	[59]	
KN1‡	γ-PV†	Skin	u. d.	
FD1‡	''	Skin	u. d.	
FS1‡	γ-PV†	Skin	u. d.	
SE87‡	''	Condyloma acuminata	[60]	
KN2‡	γ-PV†	Skin	u. d.	

*Proposed species pending on ICTV approval.

† Putative novel species.

‡ Complete genomes, not yet designated HPV numbers. SCC, Squamous Cell Carcinoma; AK, Actinic keratosis; EV, Epidermodysplasia verruciformis; u. d., unpublished data. The first row for every HPV type represents the reference clone (complete genome).

## Discussion

From a wart on the *crena ani* of a three-year-old boy we characterized the novel HPV154, which was added to the rapidly growing genus *Gammapapillomavirus*. The genome of the HPV154 demonstrated the typical genomic organization of papillomaviruses and was lacking the E5 ORF, as with γ- β- and µ-HPVs [31]. The prevalence of HPV154 was 3% in skin swabs from healthy children, which is in a similar range as other HPV types of the beta and gammapapillomavirus genera detected among adults [32]–[38]. A limitation of the study was that only samples from children were used in the screening of HPV154. Another limitation was that HPV154 was characterized from a swab sample, so we cannot assert that the virus caused the lesion. Our initial approach of obtaining the complete HPV genome by long-range PCR failed, which may be due to the low concentration of HPV154 (Table 1) and/or fragmentation of the genome. Instead we used PCRs with degenerated HPV primers and an adapted version of the DIPS-PCR method [18]. It was notable that several sequence reads of the restriction enzyme site for the adapter ligation of DIPS-PCRs could not be verified in the final sequence, which might indicate star activity of the restriction enzyme prior to ligation of adapters. Thus, compiled sequences from DIPS-PCR should be verified by independent PCR. Nevertheless, we showed that the DIPS-PCR can expand the length of sequence information, which in turns allows to design HPV primers at positions that reduce the size of the targeted long-range amplicons, which would probably increase amplification efficiency. However, our approach is laborious and time-consuming compared to modern methods such as high-throughput sequencing, and would be a feasible alternative only in cases where the latter methods are not available or other classical methods such as RCA and long-range PCR have failed.

HPV154 demonstrated the typical genomic organization of papillomaviruses, but lacked the E5 ORF as in all known members of the β- γ- and µ-PV genera [31]. The prevalence of HPV154 (5%) in skin swabs from healthy children was in a similar low range as for other HPV types of the β- and γ-PV genera detected among adults [32]–[37].

Concerning the presented phylogenetic tree, HPV154 was placed with high support along with members of the currently proposed γ-PV11 species [2]. Nevertheless, the percentages of L1 ORF sequence identity among different members of this group were closer to the interspecies center of the distribution (64.5%) than to the intraspecies center (72%) as shown in the histograms of L1 sequence identity from 189 PVs [7]. Even using 68.5% of identity as a more stringent/divergent limit for inter-species distance, three different species could be delimited, as shown with a gray dotted key in Figure 2.

Accordingly, a more stringent/divergent limit of 68.5% identity allowed us to split the proposed γ-PV11 into three putative novel species. However, the proposed γ-PV11 [2] were based on HPV types with tropism for the oral cavity, but additional HPV types within this species had been isolated from healthy skin and warts as in the case of HPV154 (Table 2). According to ICTV “A species is a monophyletic group of viruses whose properties can be distinguished from those of other species by multiple criteria” (http://www.ictvonline.org/codeOfVirusClassification.asp), but among a majority of the γ-PV species it is difficult to find distinctive characteristics besides sequence identity. Although species definition for γ-PV is currently based mainly on sequence identity [2], [6], [7], the criteria have varied over time, and hence have not been applied homogeneously to all members of the genus. For example, HPV48 (γ-PV2) shares 68.01% identity with HPV50 (γ-PV3) and 69.92% with HPV131 (proposed γ-PV14). A system with well supported phylogeny would help to define species, and it may be safer to leave new members unclassified at the species level until more isolates are sequenced.

With regard to the isolation sites enumerated in Table 2, it seems that γ-PVs can be found in a broader variety of sites than previously appreciated. Diverse sites are found for members of the same species or even type. Even though very few studies have searched for papillomavirus in the oral or nasal cavities [19], [39]–[41], members belonging to nearly every species have been identified in those studies. Altogether, this supports the idea that γ-PVs are generalist with various forms of tropism. The only exceptions seem to be the members of the γ-PV6 species, HPV101, HPV103 and HPV108, as they appear to have only mucosal tropism. This group also distinctively lack the E6 ORF and have been associated with disease [42], [43].

However, our approach to summarize isolation sites of gammapapillomaviruses described in reports provides only a suggestive mode to study the tropism of these viruses. In order to perform a rigorous evaluation of the tropism for each HPV type, a random population study with samples collected from several sites is needed.

In this study we successfully characterized the novel HPV154, thereby expanding knowledge about the diversity and tropism of HPV types in the rapidly growing γ-PV genus. In addition, we suggest that gammapapillomaviruses are generalist with broad tissue tropism. We expect that improved amplification methods will promote the discovery of additional HPV types of the γ-PV genus, and will shed light onto its apparent wider tropism compared to other papillomavirus genera.

## Materials and Methods

### Ethics Statement

Ethical approval for this study was granted by the Ethical Committee of Lund University (LU 106-01). The samples were obtained with written informed consent from the parents of the minors.

### Sample Processing: DNA Extraction

A swab suspension of 200 µl was processed using the automated MagNA Pure LC with the Total Nucleic acid kit (Roche), and eluted in 100 µl.

### Standard HPV Analysis

Sample adequacy was assessed by testing 5 µL of the sample for the human β-globin gene with a real-time PCR [44]. For identification of 22 genital HPV types (6, 11, 16, 18, 31, 33, 35, 39, 42, 45, 51, 52, 56, 58, 59, 66, 68, 70, 73, 82) simultaneously 5 µL of extracted material was added to a total volume of 25 µL for MGP-PCR and subsequent Luminex analysis [15], [16]. Five microliters was used for FAP-PCR targeting the L1 ORF [10].

### RCA

The eluted total DNA was subjected to rolling-circle amplification (RCA) using illustra TempliPhi 100 Amplification Kit (GE Healthcare), basically following the manufacturer’s instructions, but in a slightly modified form. As indicated elsewhere [45], the final concentration of each dNTP was increased to 450 µM and the reaction was incubated overnight.

### Genome Walking with DIPS-PCR

The RCA product (∼0.5 µg) was digested in order to be used as input for DIPS-PCR [18] with either TaqI, Sau3AI (Bsp143I), FatI, XbaI or HindIII enzymes (10 units, 20 µl final volume) following the manufacturer’s instructions (Fermentas). This method employs short adapters specific to each enzyme. These short oligos were synthesized to be compatible with enzymes TaqI (cgcaacgtgtaagtctg), Sau3AI (gatccaacgtgtaagtctg), FatI (catgcaacgtgtaagtctg), XbaI (ctagcaacgtgtaagtctg), and HindIII (agctcaacgtgtaagtctg); and all of them were modified to contain a phosphate group at the 5′ end and an amino group (Amino C3) at the 3′ end (MWG, Germany). The last three enzymes were added in this study in order to increase the likelihood of obtaining longer fragments and to speed up the overall process. Each of the short adapters was combined with a unique long adapter (gggccatcagtcagcagtcgtagccggatccagacttacacgttg) in equimolar quantities, to a final concentration of 25 µM. The combined oligos were incubated in a thermocycler for 4 min at 94°C, and then incubated for 5 s at 95°C. This process was repeated 18 times, reducing the temperature by 5°C in each step. The formed adapters were then aliquoted and stored at −20°C. The RCA product previously digested was combined with the appropriate (enzyme specific) double stranded adapter (50 pmol) and ligated by adding ATP (0.5 mM), DTT (10 mM) and 1.75U of ligase (Fermentas) to a final volume of 27 µl. The reaction was incubated at 16° overnight, and later diluted to 40 µl with water and was used as template for all subsequent PCR steps. Two microliters of the ligation products were subjected to an initial linear PCR amplification using a viral specific primer (0.2 µM) in 1x PCR buffer (Roche), 1.8 mM MgCl_2_, 0.2 mM each dNTP, 1.25 U of Taq polymerase (AmpliTaq Gold, Roche). The cycling conditions were 94°C 5 min; 45 cycles of 94°C 15 s, 50°C 20 s, 72° 3 min; 72° 5 min. A second PCR (exponential) using 2 µl of the linear PCR as a template was made using a nested viral-specific primer (primer sequence available upon request) combined with the adapter-specific “AP1” primer (ggccatcagtcagcagtcgtag) in the same conditions as the linear PCR but with 0.5 µM of both primers. The cycling conditions were 94°C 5 min; 35 cycles of 94°C 15 s, 55°C 20 s, 72°C 3 min; 72°C 5 min. The PCR products were evaluated by electrophoresis, and the longest product (from the different restriction enzymes) was selected, cloned using a TOPO-TA cloning kit (Invitrogen) according to the manufacturer’s instructions, and sequenced at MWG (Germany). The novel sequence was used to design primers with Oligo 7 software (Molecular Biology Insights, USA) in order to carry out successive steps of linear/exponential PCR.

### PCR with Degenerated Primers and Overlapping Amplicons

Primers were designed in the L2 ORF from a multiple alignment of HPV sequences (HPV48, 50, 60, 65, 88, 95, 112 and 116) related to the known FAP region of HPV154. The degenerated primer “L2 gamma F” (tttgratwtgaaaatcccgccttt) was used in conjunction with the HPV154 specific primer “PV77 FADI3 R” (gacctgtgctaccgactccaag). The cycling conditions were 94°C for 5 min; 40 cycles of 94°C 15 s, 52°C 20 s, 72°C 1 min and a final extension of 72° 7 min. The PCR product was purified with an Illustra Microspin S-300 HR column (GE Healthcare) and sequenced with a nested specific primer.

A second set of degenerated primers was designed for the E1 ORF. The primers ‘E1 gamma Fnew’ (gacagtggdatwkddgaagatgaa) and ‘E1 gamma Rnew’ (ttcatcttcddmwatdccactgtc), were combined with ‘FADI3 −2 nes R' and ‘FADI3 +2 nes F’, respectively (0.3 µM final concentration). One microliter of RCA-pre-amplified template was subjected to Long Range PCR (Expand Long Template PCR System, Roche) amplification in 1x PCR buffer 2 (Roche), 0.5 mM each dNTP, 0.7 U of Enzyme mix (Expand Long Template PCR System, Roche). The cycling conditions were 94°C 2 min; 10 cycles of 94°C 15 s, 50°C 1 min, 68°C 4 min and 30 cycles of 94°C 15 s, 59°C 30 s, 68°C 4 min. The product was electrophoresed and the bands excised from the agarose gel, purified with a QIAquick Gel Extraction kit (Qiagen) and cloned using a TOPO-TA cloning kit (Invitrogen), according to the manufacturer’s instructions.

The remainder of the genome was amplified using an Expand High Fidelity PCR system (Roche, Mannheim, Germany) with 2.5 µl of the RCA amplified DNA (diluted 1∶100) as a template; 0.3 µM of each primer, 3.5 mM MgCl_2_ and 1.24 U of enzyme in a final volume of 50 µl. The cycling was 94°C 2 min; 40 cycles 94°C 15 s, 55°C 20 s, 72°C 2 min increasing 5 s/cycle; 72°C 10 min. The product was purified and cloned as indicated before.

### Screening of Samples from Children

Forehead samples from children from three age groups, one month (23), one year (19), and four years old (20), were obtained from a previous study [9] and used as templates for the screening. The samples were swab suspensions in 0.9% NaCl, and were used directly as PCR templates. Primers intended for real-time PCR were designed using Oligo 7 (Molecular Biology Insights, USA) and tested for possible cross-reactivity with other types using Primer-BLAST [46]. The resulting primers were ‘HPV154 5511 F’ (accgtggtggtcctcttgg) and ‘HPV154 5647 R’ (tgggtcaaaggaaatgttttgg). The real-time PCR was carried out in an ABI 7500 Real-Time PCR Instrument (Applied Biosystems). Each test tube contained Power SYBR Green PCR Master Mix (Applied Biosystems), 0.3 µM of each primer, and 2.5 µl of template in a final volume of 25 µl. Pipetting was automated using a Qiagility device (Qiagen). The cycling conditions were 95°C 10 min and 45 cycles 95°C 15 s, 60°C 60 s, followed by melting curve analysis. For data analysis the ABI 7500 software v2.0.6 was used and the threshold for positivity was automatically calculated. The specificity of the HPV154 PCR was also analysed by gel-electrophoresis for identification of the expected 137 bp amplicon. Plasmid DNA concentration of HPV154 clone L (Figure 1) was quantified using a spectrophotometer (NanoDrop ND-1000, Nanodrop Technologies, Oxfordshire, UK). The sensitivity of the method was evaluated using serial dilutions of the clone L (Figure 1), and 10 copies were detected in a background of 1 ng of human DNA (Sigma-Aldrich, art. D 7011). The positive HPV154 control had a Tm of 75.4C (CV: 0.2%, based on four measurements).

### Viral Load of HPV154 Positive Samples

The number of viral genomes per cell was quantified by carrying out two separate quantitative real-time PCR assays to amplify a part of the HPV154 L2 gene and the human β-globin gene. For the quantification of HPV154 the primers and PCR conditions were identical to that used for screening of samples from children as described above. Quantification was extrapolated from a linear regression standard curve obtained from serial dilutions of 100,000 to 100 copies per PCR of HPV154 plasmid DNA (clone L, Figure 1) in a background of 10 ng human placenta DNA (Sigma-Aldrich, art. D 7011). The standard curve had a slope of −3.4, y intercept of 37.3 and r^2^ of 0.99. The PCR efficiency calculated from slope was 97.5%. Similarly, in order to calculate the number of cells analyzed per sample, the β-globin gene was amplified with PC03 and PC04 primers in a 25 µl PCR reaction containing 2.5 µL template [44]. The standard curve was obtained from serial dilutions of 50,000 to 50 copies per PCR of the β-globin gene using human placenta DNA (Sigma, art. no D7011). The standard curve had a slope of −3.4, y intercept of 39.9 and r^2^ of 0.99. The PCR efficiency calculated from slope was 97.5%. For the calculations of number of human cell per sample, the copy number of β-globin was divided by two. We assumed that each human cell carries two β-globin gene copies and that the diploid genome equivalent contains ∼6.6 pg DNA. No-template controls of water samples were tested of both the HPV154 PCR and the human β-globin gene PCR. All samples were analyzed triplicate. Coefficient of variation was calculated for each triplicate measurement of viral copy number per human cell. In the quantitative PCR, negative controls showed no Ct values.

### Bioinformatic Analysis

General sequence handling and feature identification were carried out using the UGENE software v1.11.5 (http://genome.unipro.ru). Putative proteins from ORFs were generated by UGENE and they were then searched for similarities with other proteins using BLASTp (http://blast.ncbi.nlm.nih.gov). Proteins were analyzed for unique domains with ScanProsite (http://www.expasy.ch/prosite) and SMART [47], including searches in the Pfam database (http://pfam.sanger.ac.se/search) [48]. A Python v3 script was made to compare pairwise identities from multiple sequence alignments (available upon request), in which all differences, including terminal gaps, were counted.

### Phylogenetic Analysis

In order to avoid missing any gamma-related virus, all complete genomes were retrieved from GenBank (914 accessions), from which 723 unique genomes remained after dereplication using Usearch v6.0.307 [49]. A workflow made using UGENE software [50] was used to extract the FAP region from L1 ORFs, align it using uMUSCLE [50], [51], and the resulting alignment was then converted to phylip format using ALTER website [52]. It was used later to make an ML-tree with RaxML (400 rapid bootstrap inferences and thereafter a thorough ML search). The branch containing all gamma-related viruses was selected using Dendroscope v3.1.0 [53] (excluding beta types). From those sequences, L1 was extracted, aligned, and dereplicated again to remove identical accessions or genomic variant sequences. The complete genomes of the remaining 91 unique sequences were manually edited to have L1 at the 3' end and then they were aligned with uMUSCLE. It was later used to make a Maximum Likelihood phylogenetic inference using RAxML v 7.4.2 [54], compiled under Linux as AVX and PTHREADS versions, and run using the raxmlGUI v1.3. Gaps were treated as missing data and the General Time Reversible (GTR) under the gamma model of rate heterogeneity was selected as a nucleotide substitution model to make 20 inferences with 150 thorough bootstrap replicates (automatically determined by the program using the majority rule tree based criteria, command “-N autoMR”). The sequence of the betapapillomaviruses HPV5 (acc. no. M17463) and HPV9 (acc. no. X74464) was used to root the tree. A graphical representation of the tree was made with FigTree software v 1.3.1 [55] and edited with Inkscape v0.48. The GenBank accession numbers of the sequences in the tree are as follows: AsPV1, HQ625440; BPV11, AB543507; BPV12, JF834523; BPV3, AF486184; BPV4, X05817; BPV5, EU360723; BPV6, AJ620208; BPV7, DQ217793; BPV9, AB331650; CG2, JF966378; CG3, JF966379; ChPV1, DQ091200; CPV13, JX141478; CPV2, AY722648; CPV7, FJ492742; DdPV1, GU117620; FA69, KC108722; FD1, JF966375; FD2, JF966376; Fi864, KC311731; FS1, JF966373; HPV4, X70827; HPV48, U31789; HPV50, U31790; HPV60, U31792; HPV65, X70829; HPV88, EF467176; HPV95, AJ620210; HPV101, DQ080081; HPV103, DQ080078; HPV108, FM212639; HPV109, EU541441; HPV112, EU541442; HPV116, FJ804072; HPV119, GQ845441; HPV121, GQ845443; HPV123, GQ845445; HPV126, AB646346; HPV127, HM011570; HPV128, GU225708; HPV129, GU233853; HPV130, GU117630; HPV131, GU117631; HPV132, GU117632; HPV133, GU117633; HPV134, GU117634; HPV135, HM999987; HPV136, HM999988; HPV137, HM999989; HPV138, HM999990; HPV139, HM999991; HPV140, HM999992; HPV141, HM999993; HPV142, HM999994; HPV144, HM999996; HPV146, HM999998; HPV147, HM999999; HPV148, GU129016; HPV149, GU117629; HPV153, JN171845; HPV154, JN211193; HPV155, JF906559; HPV156, JX429973; HPV161, JX413109; HPV162, JX413108; HPV163, JX413107; HPV164, JX413106; HPV165, JX444072; HPV166, JX413104; HPV169, JX413105; HPV170, JX413110; KC5, JX444073; KN1, JF966371; KN2, JF966372; KN3, JF966374; McPV2, DQ664501; MmiPV1, DQ269468; MusPV1, GU808564; PphPV1, GU117621; PphPV2, GU117622; PsPV1, AJ238373; RnPV1, GQ180114; SD2, KC113191; SE87, KC108721; TtPV1, EU240894; TtPV2, AY956402; TtPV3, EU240895; TtPV4, JN709469; TtPV5, JN709470; TtPV6, JN709471; TtPV7, JN709472.
